# Book Notices and Reviews

**Published:** 1865-08

**Authors:** 


### BOOK NOTICES AND REVIEWS.

*Contributions to Practical Surgery*, By W. H. VanBuren, M. D., Professor of Anatomy, University of New York. Formerly one of the Surgeons of the New York Hospital, of the Bellevue Hospital, and Vice-President of the New York Academy of Medicine, Consulting Surgeon to St. Vincent's Hospital and the Woman's Hospital, Member of the Pathological Society and the Medical and Surgical Society of New York, Member of the United States Sanitary Commission, &c , &c.

We are gratified to acknowledge the receipt of Prof. Van-Buren's contributions to Practical Surgery, issued in pamphlet form of 208 pages.

The object of the pamphlet is very concisely set forth in the preface: "The following Surgical papers have been selected from the professional periodicals in which they were orginally published, and thrown together in the present form, in the hope that they may prove useful to those interested in the pursuit of practical Surgery. Their only merit consists in the fidelity of the record they present of the results of actual practice; and the Author regrets that the unremitting claims of daily professional duty must be proffered in explanation of their obvious imperfections."

The following embrace some of the most important cases: Amputation at the Hip-joint by a modified process, the chief merit of which is rapidity of execution. "Amputation at the Hip-joint is one of the few operations in Surgery in which rapidity of execution would seem to be absolutely essential to success." The "modified process" is a combination of the circular and flap operations, is minutely and clearly described and illustrated by an elegant wood-cut.

Cases of Tracheotomy with observations. Quite a number of operations are given, which were performed for various causes, viz.: croup, foreign bodies in air passages, syphilitic ulceration of larynx, etc., inguinal aneurisms, fibrous tumor of the left ovarium, diseases of the rectum, vesical calculus, etc. The case of artero-venous aneurism with statistics is replete with useful information, especially in regard to the surgical practice which it involves.

In fact, each and every case is full of useful information of a practical character, and after a carefui perusal of them, we can honestly recommend the pamphlet to all those practising surgery, especially the junior members of the profession.

A. J. B.

---

*Annual Report of the Provost-Marshal General, Nov. 15th,* 1864. *With an Appendix embracing the Medical Report of J. H. Baxter, M. D., Chief Surgeon Provost-Marshal General's Bureau.*

Surgeon Baxter has placed the medical profession under lasting obligation in contributing to medical science, such a variety of invaluable statistical information concerning the diseases and infirmities of mankind.

If space would admit we would gladly reproduce the entire series of tables, 22 in number. The Report is an elaborate and carefully condensed document, and is an honor to the Bureau from which it proceeds. Every page attests to the ability, industry and conscientiousness of the compiler. It is a national record, and as each succeeding year carries us farther and farther from the war, periods of prosperous commerce and peaceful civic employments preparing the way for the advent of the Great American Historian, who is yet to come, and narrate faithfully and with a graphic pen, the record of the tremendous Revolution through which we have so triumphantly passed, this Report, and those which have preceded it, will be of inestimable value.

We cannot conclude our brief notice of this important document without adding our mite to the general meed of praise so freely awarded to Surgeon Baxter, wherever his name and services are known. His knowledge of his profession is only equalled by his executive ability, and those courteous and kind actions which ennoble and befit a gentleman and an American officer.

F.

*Graduates of Medical Colleges, Session of* 1864–5.—Jefferson Medical College, 136; University of Pennsylvania, 117; Rush Medical College, 108; Bellevue Hospital Medical College, 93; College of Physicians and Surgeons, 79; Long Island College Hospital, 52; University of Michigan, 51; Buffalo Medical College, 50; Ohio Medical College, 46; Albany Medical College, 45; Massachusetts Medical College, 42; Charity Hospital Medical College, 38.

---

MARRIED.—In Wyanet, Ill., on the 15th of July, by the Rev. Wm. Foreman, F. C. ROBINSON, M. D., to Miss MARY E. HULL, both of that place.

At the residence of the bride's parents, July 11th, by Rev. Isaac Linebarger, A. M., GEORGE W. BEGGS, M. D., of Naperville, and Miss LILLA SIMS, of Plainfield.

---

*Gen. Tom Thumb, his Wife and Baby.*—Mr. and Mrs. Stratton, (General Tom Thumb and his wife) have been giving receptions at Calcaldi's Hotel, Dover street, Picaadilly. The General looks remarkably well, and has improved in appearance since we last saw him. He is now twenty-seven years old. His wife is smaller than the General, is dark, with very well-defined features, indeed exceedingly pretty, good figure, and inclined to *embonpoint*. Her age is twenty-three. The baby was also exhibited, which is now twelve months old, weighing seven pounds and three quarters. The diminutive pair seem very proud of their offspring; whether it will be of the same lilliputian stamp we cannot at present say. Mrs. Stratton's sister, aged twelve, was also present. She is smaller still than the General or his wife.—*Lancet.*

